# *In Vivo* and *In Vitro* Assessment of Human Saphenous Vein Wall Changes

**DOI:** 10.2174/1874192400701010015

**Published:** 2007-08-17

**Authors:** Akram M Asbeutah, Sami K Asfar, Hussain Safar, Mabayoje A Oriowo, Ihab ElHagrassi, Mona A Abu-Assi, James D Cameron, Barry P McGrath

**Affiliations:** a Department of Medicine, Monash University and Department of Vascular Sciences in Southern Health, Dandenong Hospital, Melbourne, Australia; b Department of Surgery, Faculty of Medicine, Kuwait University and Department of Surgery-Vascular Unit, Mubarak Al-Kabeer Hospital, Kuwait; c Department of Pharmacology, Faculty of Medicine, Kuwait University, Kuwait; d Department of Cardiovascular Diseases, Chest Hospital, Kuwait; e Department of Science, School of Basic Education, Public Authority of Applied Education & Training, Kuwait

**Keywords:** Primary varicose veins, chronic venous insufficiency, noradrenaline, 5-hydroxyptamine, *in vivo* vein study, *in vitro* vein study

## Abstract

**Purpose:**

To investigate if noradrenaline (NA) and 5-hydroxyptamine (5-HT) drugs induce responses of isolated control and varicose veins are altered by removal of the endothelium.

**Subjects & Methods:**

Specimens of the great saphenous vein (GSV) were obtained from 12 subjects with primary varicose veins and 12 subjects from donor vessels at cardiac surgery. A total of 10 normal healthy volunteers were selected for comparison. The diameter changes of GSV during the resting phase, at the end of 5 minutes occlusion, and then every 30 seconds post deflation for five minutes were measured using B-mode ultrasound. Post-surgery the vein sample was collected in a tube of Krebs-Henseleit solution.

**Results:**

The repeated measure ANOVA test for the diameter, percent, and difference changes of GSV diameter from maximum diameter at different time intervals showed significance difference within and between all groups. NA and 5-HT produced concentration-dependent contractions of control and varicose saphenous vein segments. There was no significant difference in the potency of NA and for 5-HT, but the maximum response, normalized for tissue weight, was less in varicose vein segments. Removal of the endothelium had no effect on the potency of NA or 5-HT but significantly (p<0.05) reduced the maximum response to NA and 5-HT in varicose vein segments but not to 5-HT in control veins.

**Conclusion:**

The venous endothelial damage may cause vascular smooth muscle contractions dysfunction that favours dilatation and secondary valvular insufficiency.

## INTRODUCTION

There is some controversy regarding the primary pathology that leads to the development of venous incompetence. Various theories, the valvular hypothesis [1] and the vein wall hypothesis [2], have been put forward to explain the pathogenesis of varicose veins and chronic venous insufficiency (CVI). Although the pathophysiology of the disease is largely unknown, abnormalities of the venous wall and the valves are considered to play a major role [3]. The cause of this abnormality has been assumed to be the result of either a defective smooth muscle function or connective tissue damage [4] resulting in distension, deficient leaflet apposition and lack of closure rather than primary valve damage. A general weakness of venous smooth muscle does not seem to be involved since the defect is localized and proportional to wall deformation [4]. However, Thulesius reviewed the four most important etiological factors which can be involved in the development of CVI [5].

Patients with varicose veins exhibit increased threshold autonomic nerve function to temperature and vibration stimuli [6]. Reduced contractile responses to noradrenaline [7] and imbalance between endothelial cell-derived contractile and vasodilator responses [8] have been described for varicose veins. Hypothermia cause vasoconstirction of normal veins while varicose veins are not affected [9]. Also Thule-sius found in normal veins no endothelial-derived nitrous oxide (NO) relaxation upon acetylcholine stimulation, whereas NO was released from the endothelium of varicose veins [7]. However, capacitance veins have been shown to both release basal and stimulated NO [10]. Moreover, the reduced tone in varicose veins and loss of endothelial derived contracting factor (EDCF) may be related to a primary endothelial abnormality since ultra-structural studies have shown endothelial damage in varicose veins [11, 12].

Investigators have concentrated on the collagen, elastic, and muscle fiber components of the vein wall and their effect on the overall physical properties of the conduit [13]. Abnormalities in these components are certainly important but the endothelial lining of the vein wall and flow properties of the blood within these have received much less attention [14–16].

The aim of the *in vivo* phase of the study was to examine vein function by measuring diameter changes of the great saphenous vein (GSV) using duplex ultrasound (DUS) before, during, and after venous occlusion in control, normal, and varicose veins subjects. In the second *in vitro* phase of the study the aim was to investigate if noradrenaline (NA) and 5-hydroxytryptamin (5-HT) induce responses of isolated control and varicose veins that were altered by removal of the endothelium.

## METHODS

### Subjects

Subjects were recruited from the general population prior to varicose vein surgery (varicose vein group) and undergoing coronary artery bypass graft (CABG) but without varicose veins (control group). A total of 24 venous segments specimens were studied. Varicose vein subjects undergoing surgery for stripping the great saphenous vein were asked to be off all venotropic drugs including Daflon 500 mg. Control subjects undergoing CABG were required to have no varicose vein or any evidence of venous insufficiency. Ten normal volunteers without any history of diseases or medications were matched for age and sex for comparison.

Subjects were asked to complete a questionnaire administered by the author that included personal and occupational details, relevant medical and family history, and possible risk factors for venous disease. They underwent duplex ultrasound to diagnose venous reflux and the great saphenous vein, above knee, was assessed for diameter changes in response to occlusion-release stimulation, and was marked one day before surgery by permanent pen, so that the vein segment could be harvested for *in vitro* part of the study. The study was approved by the Human Research and Ethics Committee from Kuwait University, Faculty of Allied Health Sciences and Ministry of Health, Mubarak Al-Kabeer Hospital at Kuwait. All subjects were asked to sign the consent form.

### Duplex Ultrasound Scanning (DUS) Techniques- *In Vivo* Part

All subjects were assessed in temperature-controlled (22±2°C) environment using a Philips HDI 5000 ATL (Phil-ips Medical Systems, Bothell, WA) ultrasound machine equipped with linear 7-4 MHz transducer. Venous reflux was elucidated by manual compression distal to venous segments under examination. Venous reflux duration more than one second is considered abnormal [17]. Firstly, venous reflux was assessed in all subjects in standing position while they were holding to the edge of a couch or frame and the weight distributed in the other leg. Measurements were made along the deep and superficial veins of leg with varicose veins (varicose vein group), veins of subject legs without varicose veins prior to GABG (control group), and normal legs (normal group). The venous reflux procedure was similar to the previously described method [17, 18].

Subject was then placed in a supine position with the leg to be investigated elevated 30cm at the heel. The knee was slightly flexed and externally rotated while ensuring adequate relaxation of the leg. After 20 minutes rest the diameter of the great saphenous vein 4 cm above the kneecap was measured and then venous occlusion was induced by inflation of a pneumatic cuff (17 cm) placed around the upper-mid thigh to a pressure of 80 mmHg until the maximum diameter is achieved. When there was no further changes in the diameter (approximately 5 minutes), the cuff was deflated. The diameter of the vein was measured every 30 seconds for the first two minutes and then every one minute for five minutes. The great saphenous vein diameter was measured with B-mode ultrasound during quite respiration. In applying the transducer against the cutaneous surface, minimal pressure was used to guarantee satisfactory saphenous vein imaging, while avoiding undesirable compression that could have modified vein diameter assessment. This was easily obtained in every case, since vein compression is clearly visible on the ultrasound image. In measuring vein diameter the axis parallel to the skin was preferred as it depends more on the flattening of the saphenous vein [19]. Venous diameters were measured from wall to wall in longitudinal section 4 cm above the kneecap base using the electronic callipers incorporated in the duplex ultrasound machine software. Once satisfactory position was found, the investigator requested the subject to maintain that same position throughout the study. Diameters were determined where the walls were best visualized, ignoring luminal irregularities.

Following studies of *in vivo* venous responses to occlusion and reperfusion, surface skin markings were made 4 cm about the knee in 12 of the legs exhibiting varicose veins and in 12 of the normal legs of control subjects awaiting CABG surgery. At surgery the marked great saphenous vein segments were harvested for subsequent *in vitro* pharmacological studies.

### Vein Harvesting & Pharmacological Study- *In Vitro* Part

The specimens were harvested at operations for radical removal of varicose veins under pentobarbital-halothane or spinal anesthesia. Moreover, the anesthesia for control: di-azepam 100 μ/kg, papaveretum 120 μg/kg, fentanyl 40 μg/kg and pancuronium bromide 100 μg/kg.

After excision the vein samples were put into cool Krebs-Henseleit solution and send for testing immediately or kept in a fridge at 4°C for testing within 1-4 hours of sample collection. The specimen was cleaned of connective tissue and fat and cut into two rings, each 4 mm in length. During this procedure great care was taken to avoid unintentional rubbing of the intimal surface so as to maintain the integrity of the endothelial layer and meanwhile not damaging the muscular layer of the vessel. One ring served as control and the other was de-endothelialized by inserting the tip of a small forceps and gently rolling the ring for 15 seconds according to the method of De May and Vanhoutte [20]. The preparations were vertically mounted in 25 ml organ bath filled with Krebs-Henseleit solution (NaCl 113 mmol/L; KCl 4.6 mmol/L; CaCl_2_ 2.5 mmol/L; MgSo_4_ 1.2 mmol/L; NaHCO_3_ 22.1 mmol/L; KH_2_PO_4_ 1.1 mmol/L and glucose 11.0 mmol/L) maintained at pH 7.4, at a temperature 37°C and gassed with 95% oxygen and 5% carbon dioxide.

**Fig. (1):**
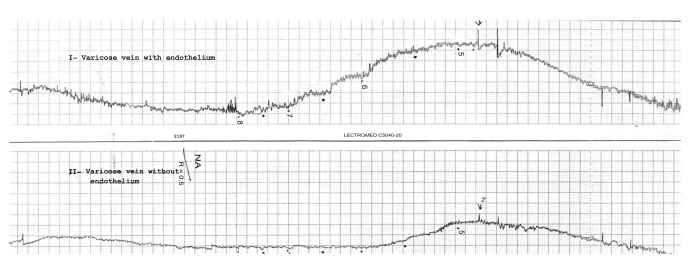
Shown here are contractile responses of two rings of a varicosed human saphenous vein preparation recorded simultaneously. One rings with endothelium (I) and the other de-endothelialized (II). First part: washing and stabilizing with 80 mM KCL solution; and second part: dose-response to increasing contractions of NA.

The vein rings were attached to the bottom of the organ bath and the upper end connected to dynamometer UF-1 force transducer. Isometric contractions were continuously recorded on a Lectromed 4-channel polygraph (MultiTrace 4P). Before the experiments were begun a pretension of 2 gram was applied to the preparation by adjustment of a micrometer screw. The system was allowed to equilibrate for 30-40 minutes. After the period of equilibration, KCl (80 mM) was added to the bath to test for tissue viability. This concentration of KCl was repeated after 30 minutes. The tissues were then washed repeatedly over the next 30 minutes period. Thereafter, agonist (NA or 5-HT) concentration-response curves were established by adding, cumulatively, increasing concentrations (0.5 log unit increments) of each agonist to the bath and allowing the response to each concentration reach a peak before adding the next concentration. In all cases, two consecutive concentration-response curves were separated determined by a rest period of 60 minutes. The second concentration-response curve was used as the control (Fig. 1). Agonist potencies were expressed as pD_2_ values, where pD_2_ is the negative logarithm of the agonist concentration producing 50% (EC50) of the maximum response. EC50 and maximal contraction were determined from dose-response curves.

Rubbed and non-rubbed rings from the same vessel were subjected to identical testing steps which were run and recorded simultaneously in two organ baths. Similarly, vaso-constrictor drug effect in control veins was tested with rubbed and non-rubbed endothelium. The experiments were repeated using the drug 5-HT on varicose and control vein segments from different subject. At the end of the experiments, the venous segments were blotted and weighed. Maximum response was expressed as mg/mg tissue weight.

The ultrasound measurements were performed by the author. Data was stored into the hard disks of the ultrasound machine and any data from any former ultrasound measurement was not examined prior to the next diameter measurement. The pharmacological tests were performed with the help and supervision of one pharmacology technologist.

### Statistical Analysis

Data was entered and analyzed using the SPSS version 12 for Windows. Data are reported as mean±SD. The multiple repeated measurement (ANOVA) was used to determine the significance of difference between control, normal, and varicose veins (percent diameter changes from maximum diameter at occlusion and difference diameter changes from maximum at different time intervals). Cross-tabulation analysis (Chi-Square test) was used to test for the significance of the differences among groups in return of vein diameters to<10% of resting baseline.

Data was recorded as mean of ‘n’ experiments where n is the number of samples from ‘N’ subjects. Graphs were plotted and analyzed using Graphpad Prism software. Differences between mean values were tested for significance using paired Student’s *t*-test. The difference was assumed to be significant when p<0.05. The relationship between duplex ultrasound and pharmacological parameters were carried out using Pearson correlation coefficients test.

## RESULTS

### Subject Characteristics

Specimens of the great saphenous vein were obtained from 12 subjects with primary varicose veins above knee (6 females and 6 males, aged 41.4±8.5 (mean±SD year), and with body mass index (BMI) 27.1±4.5 (mean±SD kg/m^2^). Control great saphenous vein specimens were obtained from donor vessels of the great saphenous vein above knee at cardiac surgery from 12 subjects (6 females and 6 males, aged 50.2±8.3, (mean±SD years) and with BMI 29.9±4.5 (mean±SD kg/m^2^). Table 1 summarize population characteristics. Table 2 summarizes subject’s medical conditions and medication history.

### *In Vivo* Duplex Ultrasound Findings

Table 3 presents the mean, percent, and difference of diameter changes of great saphenous vein from maximum diameter during the resting phase (pre-occlusion), at the end of 5 minutes occlusion, and then every 30 seconds post deflation for the first two minutes, and every 1 minutes until five minutes post deflation for 12 control, 10 normal, and 12 varicose vein segments.

The control venous segments average resting diameter was 0.42±0.06, for normal venous segment was 0.4±0.06, and for varicose veins was 0.42±0.11. Average maximum dilation diameter after 5 minutes of occlusion was 0.55±0.06 in control, 0.52±0.06 in normal, and 0.58±0.12 in varicose vein segments, and diameter alteration had returned to 10% original resting diameter in 9 out of 12 control veins, 10 out of 10 normal veins, and 4 out of 12 varicose veins segments within 30 seconds of deflation but the remaining 3 control and 8 varicose vein segments the diameter alteration had still not returned to resting within more than 60 second of deflation (Cross-tabulation analysis). In addition, the cross-tabulation analysis were carried out for 20% and 30% and found to be significant but not at 30% for all groups. It is apparent that the varicose vein segments dilate more and return more slowly to original resting diameter than the control and normal venous segments.

The repeated measure ANOVA test for the diameter, percentage, and difference changes of great saphenous vein diameter from maximum diameter at different time interval showed significance difference (p <0.05) within and between control, normal and varicose vein venous segments. Data are represented in Table 3.

### *In Vitro* Pharmacological Findings

**Fig. (2):**
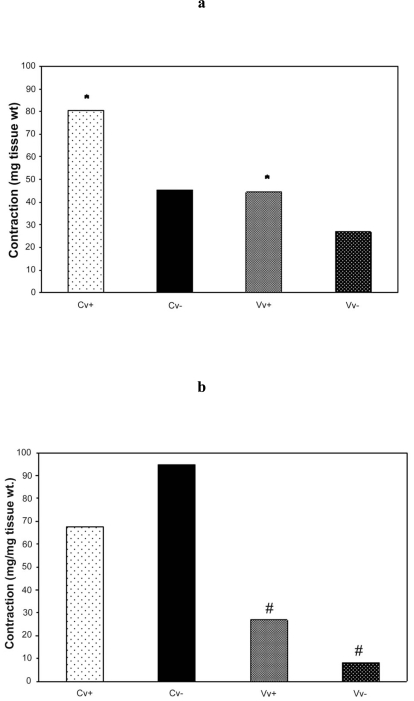
Bar graphs of human great saphenous vein rings to increasing concentration of (**a**) NA and (**b**) 5-HT for control and varicose vein segments. Endothelium (+) and rubbed (-) in each control (Cv) and varicose veins (Vv) specimens. ^*^p<0.05 Cv+ compared to Vv+ and **^#^** p<0.05 Vv+ compared to Vv- using Student’s paired *t*-test.

NA and 5-HT produced concentration-dependent contractions of control and varicose saphenous vein segments. There was no significant difference in the potency of NA (log EC50 values were -5.55±0.06 and -5.58±0.10, n =6, in control and varicose vein segments, respectively) and for 5-HT (log EC50 values were -6.44±0.08 and -6.66±0.09, n=5, in control and varicose vein segments, respectively) between the two groups but the maximum response, normalized for tissue weight, was less in varicose vein segments for both NA and 5-HT. Results are represented in Fig. (2**a**,**b**) and Table 4.

Removal of the endothelium significantly (p<0.05) reduced the maximum response to NA in control veins. The maximum response to NA in varicose vein segments was not statistically significant after endothelial denudation. Removal of the endothelium had no effect on the potency of 5-HT in control vein segments but significantly (p<0.05) reduced the potency of 5-HT in varicose vein segments (log EC50 values were -6.66±0.09 and -6.13±0.10, n=5, in vein segments with and without the endothelium, respectively). Results are represented in Fig. (2**a**,**b**) and Table 4.

**Fig. (3):**
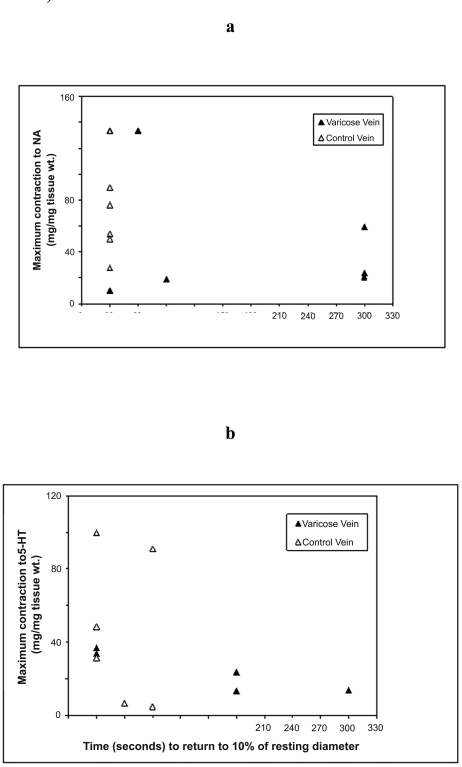
Correlations of the maximum contraction responses of varicose and control vein segments as measured *in vitro* for (**a**) NA; and (**b**) 5-HT to time to return to 10% of resting diameter as measured *in vivo*.

**Fig. (4):**
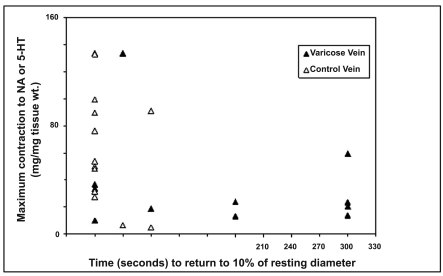
Relationship between maximum contraction responses of varicose and control vein segments as measured *in vitro* with NA or 5-HT and time to return to 10% of resting diameter as measured *in vivo.*

The relationships between in vivo and in vitro responses were examined and correlations are shown in Figs. (3**a**,**b**) and (4). Whilst there was a trend for an inverse relationships between the maximum contraction responses to NA, 5-HT and the rate of contraction/return to resting diameters of the same venous segments in vivo, the results did not achieve statistical significance. The (r) values for 5HT responses and combined 5HT and NA responses were -0.32 and -0.40 respectively.

## DISCUSSION

The main findings of the ultrasound component of the study were that the control and normal group showed almost identical responses. This suggests that the venous wall was functioning normally in the control group. In contrast, in the varicose vein group the vein dilated more and returned more slowly to its original diameter. A weaker maximal contraction in varicose veins when compared to control veins was expected and this was confirmed in this study.

The findings of the present study must be taken in context with the subjects investigated and the methods of investigations. The former is important because the control group subjects had underlying risks for cardiovascular diseases as from Table 2 it is clear that the majority of subjects from which the veins were taken had hyperlipidemia, diabetes or both. Furthermore they were, as would be expected, on multiple drugs including a large number on nitrates which could clearly have an effect on their responses but, more importantly, they did not have any history of CVI as investigated clinically and by ultrasound. To overcome this problem, healthy volunteers without any venous diseases or any medications history were entered to our present study to correlate the diameter changes by ultrasound to control group versus the varicose vein group. The method of investigation is also important because it may affect the sensitivity of the outcome. The ultrasound diameter measurements in all subject groups were assessed by the same experienced vascular technologist (A.A) in a room at constant temperature (22±2°C).

The pharmacological study confirmed the findings of Thulesius [7] in that there was contractile reduction in the maximal contractile force of varicose vein preparations to NA and 5-HT compared to controls. This was more evident when the endothelium was denuded. The veins were de-endothelialised by using the standard technique of De May and Vanhoutte [20]. Moreover, endothelial denudation increased the maximum response to 5-HT in control vein segments but this was not statistically significant. This data could be explained by a lack of release of endothelial derived relaxing factor(s) (EDRFs), in particular NO as was suggested by Datte and colleagues in studies of isolated rat portal vein segments [21]. However, the results must be interpreted with caution given the large variation in contractile responses of the vein segments, possibly influenced by stretch or damage to the tissue during surgery.

The reason for reduced contraction of varicose veins may involve disordered smooth muscle responsiveness and/or endothelial dysfunction. The reduced contractile responses to NA suggest the former; the responses of GSV segments from subjects with varicose veins to 5-HT, showing a further decrease in maximum contraction after endothelial denudation, in contrast to the control vein responses, suggest the latter. It is possible that the significant fall in 5-HT induced maximum contraction of the varicose vein rings after endothelial denudation may reflect an endogenous endothelial-dependent venoconstrictor influence in these rings, perhaps due to en-dothelin.

By correlating the ultrasound findings with the pharmacological findings, it might be considered that dilatation of the varicose vein segments with slow return to initial resting diameter represents a contraction dysfunction of the venous wall initiated most probably by the endothelium due to deficient EDCF or due to a release of EDRF in a healthy vascular smooth muscle fibres in the venous wall of the varicose vein group. The results of a previous study [22] clearly demonstrated intimal hypertrophy and hyperplasia of the suben-dothelial smooth muscle cells in saphenous varicose vein samples. Also they reported that the changes in the subendo-thelial smooth muscle cells did not correlate with any changes in the endothelial cells of the varicose veins studied. They suggested that the endothelial cells do not go through the same cascade of events that the smooth muscle cells undergo. Despite the fact that endothelial cells not only act as barriers but also as regulators of vascular tone [23]. Nevertheless, it has been reported that a significant increase in endothelial adhesion molecule ICAM-1, associated with more thromboxane A_2_ and prostaglandin E_2_ synthesis, and with an increased interaction among leukocytes, platelets and endothelial cells, is observed in varicose veins [24, 25]. This could imply that although the morphology of endothelial cells may not change in varicosis, the overall function of such cells may. In the present study, the combination of structural and functional differences between normal and varicose GSVs defined by ultrasound, with differences in *in vitro* contractility of the same segments, suggest that endo-thelial dysfunction may be an important aetiological factor in the pathogenesis of primary varicose veins and CVI development.

Whilst there was a trend for the maximum *in vitro* veno-constrictor responses to be inversely related to the *in vivo* functional responses (time of return of vein within 10% of its resting diameter), with (r) value of -0.32 for all data and -0.40 for 5-HT responses, these do not reach statistical significance. The significant variability of responses suggests that greater numbers need to be studied to further explore this relationship.

Finally, in order to come up with a proper management of varicosis, it is essential to understand the basic mechanisms leading to the development of such a disease in people at various ages. The prevailing view of the aetiology of varicose veins revolves around the trigger(s), as well as the cascade of events. A better understanding of the nature of the triggers(s) and of the progressive changes occurring in the vessel wall of the varicose vein, obtained by combining structural and functional studies, including electron microscopy as well as pharmacological techniques, may lead to not only a better treatment, but also prevention of the disease. Also studies are necessary to explore haematological as well as biochemical markers in varicose veins subjects, and how these change with disease development. In view of that en-dothelial damage may cause vascular smooth muscle dysfunction that favours dilatation and secondary valvular insufficiency, it could equally well be that valvular insufficiency leads to dilatation which causes endothelial damage. Our present studies do not allow differentiation between these two possibilities and further studies are needed to resolve such issue. In addition the role of calcium storage in vascular smooth muscle in the wall of the veins warrant more study.

## CONCLUSION

Varicose venous segments dilated more during occlusion hyperemia and returned slowly to their original resting diameter when compared to control and normal venous segments. This might be attributed to venous wall dysfunction. Venous endothelial damage may cause vascular smooth muscle contractile dysfunction that favours dilatation and secondary valvular insufficiency with deficient cusps apposition or valvular insufficiency leads to dilatation which causes endothelial damage. This may be the primary factor in the development of varicose veins and CVI.

## Figures and Tables

**Table 1:** Subject Characteristics

Characteristic	Control	Normal	Varicose Veins
Subject & Limb numbers	12	10	12
Age (mean±SD, years)	50.2±8.3	38.7±7.7	41.4±8.5
Sex (Number, M/F)	6/6	5/5	6/6
Body Mass Index (mean±SD, kg/m^2^)	29.9±4.5	28.1±1.8	27.1±4.5

**Table 2:** Subject Medical Conditions and Medications

Characteristic	Control Veins (N)	Varicose Veins (N)
**Medical Conditions**		
DM, HT, HL, & MI	5	3
DM, HT, & MI	3	0
DM, HL, & MI	3	0
DM & HT	4	0
HT	0	2
HT & HL	4	0
HT, MI, & CVA	2	0
HT & MI	3	0
DM & MI	1	0
**Medications**		
Aspirin	24	3
Isosorbide dinitrate	17	3
Atenolol	10	2
Metroprolol	10	2
Propranolol	1	0
Amlodipine	5	3
Diltiazem	3	0
Captopril	4	0
Lisinopril	2	0
Frusemide	4	0
Atorvastatin	6	0
Simvastatin	4	0
Micronized flavonoid (diosmin 90% and hes- peridin 10%)	0	2

DM= diabetes mellitus; HT=hypertensive; HL= hyperlipidemic; MI= myocardial infarction; CVA= cerebrovascular accident; and N= number of subjects.

**Table 3:** Mean Vein Diameter (M), Percent (%) Diameter Changes from Maximum, and Difference (D) Diameter Changes from Maximum Data for Control, Normal, and Varicose Vein Segments

Time		Control Veins	Normal Veins	Varicose Veins
Resting	M	0.42±0.06	0.40±0.06	0.42±0.11
	%	76±5	77±6	72±11.5
	D	-0.13±0.03	-0.12±0.03	-0.16±0.08
Maximum	M	0.55±0.06	0.52±0.06	0.58±0.12
At 300s	%	100	100	100
Occlusion	D	0	0	0
30s post	M	0.44±0.06	0.43± 0.05	0.48±0.11
Deflation	%	80±5.5	82±4	82±8
	D	-0.11±0.03	-0.09±0.02	-0.10±0.06
60s post	M	0.44±0.06	0.41± 0.05	0.48±0.11
Deflation	%	80±5.2	79±6.7	82±9
	D	-0.11±0.03	-0.11±0.04	-0.10±0.06
90s post	M	0.43±0.06	0.41± 0.05	0.46±0.11
Deflation	%	78±5.3	79±7	79±9
	D	-0.12±0.03	-0.11±0.04	-0.12±0.06
120s post	M	0.43±0.06	0.40± 0.05	0.46±0.11
Deflation	%	78±5.3	77±7	79±9
	D	-0.13±0.03	-0.12±0.04	-0.12±0.06
180s post	M	0.43±0.06	0.40± 0.05	0.45±0.11
Deflation	%	78±5.7	77±6.3	77±9.5
	D	-0.13±0.03	-0.12±0.04	-0.13±0.07
240s post	M	0.43±0.06	0.40± 0.05	0.45±0.11
Deflation	%	78±5.4	77±6.3	77±9.5
	D	-0.13±0.03	-0.12±0.04	-0.13±0.07
300s post	M	0.43±0.06	0.40± 0.05	0.45±0.11
Deflation	%	78±5	77±6.3	77±10
	D	-0.13±0.03	-0.12±0.04	-0.13±0.07

Values are mean±SD of diameter for all subject groups. ANOVA test showed significance within and between all groups at different time interval (p<0.05).

**Table 4:** Contractile Effect (Mean±SEM) of NA and 5-HT on Control and Varicose Vein Segments (+) with Endothelium; (-) Without Endothelium; (Cv) Control Vein Specimens; (Vv) varicose vein specimens; N = Number of Specimens; EC50 = 50% Effective Concentration; Max Cont = Maximum Contraction mg/mg Tissue Weight; * p<0.05 Cv+ Compared to Vv+; ^#^ p<0.05 Vv+ Compared to Vv- Using Paired Students’ *t*-Test

	Noradrenaline			5-Hydroxytryptamine		
	log EC50	Max Cont	N	log EC50	Max Cont	N
**Cv+**	-5.55±0.06	80.62±15	6	-6.44±0.08	67.63±16.41	6
**Cv-**	-5.71±0.08	45.42±11.4	6	-6.30±0.14	94.87±34.26	6
**Vv+**	-5.58±0.10	44.4±19.2*	7	-6.66±0.09	26.88±5.32	5
**Vv-**	-5.58±0.08	26.9±9.3	6	-6.13±0.10^#^	8.08±4.33^#^	5
